# Positive and Negative Affect Differentially Predict Individual Differences and Intra-Individual Changes in Daily Cognitive Failures in Younger and Older Adults

**DOI:** 10.3390/brainsci14121259

**Published:** 2024-12-15

**Authors:** Ysabel A. Guevarra, Nadyanna M. Majeed, Eva M. Hisham, Andree Hartanto

**Affiliations:** 1 National Institute of Education, Nanyang Technological University, 1 Nanyang Walk, Singapore 637616, Singapore; 2 School of Social Sciences, Singapore Management University, 10 Canning Rise, Singapore 179873, Singapore; 3 Faculty of Arts and Social Sciences, National University of Singapore, 5 Arts Link, Singapore 117570, Singapore

**Keywords:** positive affect, negative affect, cognitive failures, daily diary, multilevel modelling

## Abstract

(1) Background: Cognitive failures, including lapses in attention, memory, and executive functioning, can negatively affect daily performance and well-being. Negative and positive affectivity have been implicated in cognitive functioning, yet their relationship with cognitive failures remains underexplored. This study investigates the impact of positive and negative affect on cognitive failures, using daily diary methods to examine both within-person and between-person associations in a sample of younger adults from Singapore and adults across the lifespan from the United States (US). (2) Methods: Participants (Singapore: *N* = 253, US: *N* = 1726) completed daily diaries over seven (Singapore) or eight (US) consecutive days. Multilevel modelling was used to analyse both within- and between-person relationships between affect and cognitive failures, controlling for demographic and socioeconomic variables. (3) Results: In both the Singapore and US samples, negative affect was consistently positively associated with cognitive failures at both levels (SG within-person: β = 0.21, *p* < 0.001; SG between-person: β = 0.58, *p* < 0.001; US within-person: β = 0.08, *p* < 0.001; US between-person: β = 0.28, *p* < 0.001), supporting the influence of negative affective experiences on cognitive lapses. However, positive affect showed no significant associations with daily cognitive failures in the Singapore sample (within-person: β = 0.01, *p* = 0.683; between-person: β = −0.04, *p* = 0.484) and only a between-person negative association in the US sample (within-person: β = 0.02, *p* = 0.157; between-person: β = −0.11, *p* < 0.001). (4) Conclusion: These findings suggest that positive and negative affect differentially influence individual differences and intra-individual changes in daily cognitive failures among both younger and older adults.

## 1. Introduction

Cognitive failures—the inability to complete routine or simple tasks that an individual typically performs with ease [1]—are generally the result of lapses in attention or memory. Individuals who experience cognitive failures frequently report recurring errors in action, perception, and memory [2]. These cognitive lapses are not only common [3,4] but can also have significant consequences for daily functioning. Whether forgetting to complete a task, misplacing items, or losing focus during important activities, cognitive failures can hinder productivity and decision-making. For instance, cognitive failures have been shown to increase the likelihood of driving accidents and violations [5,6,7] as well as the risk of workplace accidents and injuries [8,9,10]. The prevalence and potential risks of these lapses highlight the importance of understanding their underlying causes, given their detrimental effects on well-being and overall quality of life [3,11].

Given the importance of cognitive failures in everyday life, extensive research has focussed on identifying environmental factors that significantly contribute to these lapses. Studies have explored various situational influences, such as technological factors like smartphone use [12,13,14], and exposure to stressors [15,16], including discrimination [17,18] and chronic work stress [19,20]. These experiential factors are thought to deplete an individual’s limited cognitive resources, reducing the cognitive capacity available for current tasks [21]. However, despite the substantial research on environmental factors, there has been limited attention to emotional factors, such as affective states, which may play an important role in influencing cognitive failures.

Several theoretical perspectives provide insights into how affective states might influence cognitive failures. Positive affect has been shown to broaden attentional scope, facilitating flexible thinking and global processing, while negative affect narrows attentional focus, enhancing cognitive control and local processing [22,23,24]. This aligns with the level-of-focus hypothesis [25], which posits that emotions influence the breadth of cognitive processing by directing attention to different aspects of stimuli. Specifically, positive affect promotes broader attention to both the task at hand and unrelated stimuli, as it evokes a sense of security that does not necessitate focussed attention, while negative affect directs attention to specific, local details, signalling potential threats that require detailed processing [26,27]. These perspectives are further supported by Fredrickson’s broaden-and-build theory [28], which suggests that positive emotions expand attentional scope, facilitating greater cognitive flexibility and cognitive resources, but with less depth of attention provided to tasks. Conversely, the theory also posits a “narrow hypothesis,” whereby negative emotions constrict attention due to their association with specific action tendencies, such as “fight or flight”. Together, these theoretical frameworks suggest that affective states play a critical role in cognitive failures. Positive affect may inadvertently increase cognitive failures by broadening attention, which decreases the depth of focus and incorporates task-irrelevant stimuli [29,30], while negative affect may reduce cognitive failures by promoting controlled and focussed attention [31].

Other studies have shown that, in certain situations, positive affect may narrow attentional focus while negative affect may broaden attentional scope. For example, during stressful life events related to economic challenges or COVID-19 stressors—both linked to negative affect—individuals often experience higher cognitive failures [32,33]. This may occur as attention shifts toward irrelevant stimuli, such as job insecurity or health concerns, rather than remaining on daily tasks. One explanation for this phenomenon is the mediating role of mind wandering, which involves thoughts about personal goals unrelated to the task at hand [34,35,36]. Stress-induced mind wandering can impair cognitive performance, leading to lapses in attention and increased cognitive failures [37,38,39]. Conversely, other studies have shown that inducing positive affect can enhance cognitive performance [40,41,42], which may result in fewer cognitive failures [4,43,44,45]. Thus, it is also plausible that positive affect decreases cognitive failures, while negative affect increases them. These conflicting theoretical predictions underscore the need to clarify how both positive and negative affect influence cognitive failures, particularly in daily contexts.

Thus, this study aims to examine daily affect, as operationalised by end-of-day assessments of positive and negative affect [46], and its effect on daily cognitive failures at both the within-person and between-person levels using data from two daily diary studies—one for seven days (Singapore sample) and one for eight days (US sample). Utilising the daily diary method provides the unique advantage of capturing affective states and cognitive lapses in a naturalistic setting in real-time, minimising time lag and reducing recall bias [47,48]. Tracking participants over a week was considered the most appropriate approach because the variables of interest, such as daily affect and daily cognitive failures, are short-term and individual-based experiences [49]. Given the frequent occurrence of these variables, collecting data once daily for a week provides an ideal representative sample, capturing multiple observations of the constructs [50]. Taken together, based on the level-of-focus hypothesis [25] and broaden-and-build theory [28], we hypothesise that individuals with higher positive affect at both the within-person and between-person levels will experience more cognitive failures daily, as positive emotions point to a wider attentional scope that directs attention to task-unrelated stimuli. Conversely, we predict that individuals with higher negative affect at both the within-person and between-person levels will experience fewer cognitive failures due to a more controlled, narrowed attentional scope.

## 2. Materials and Methods

### 2.1. Transparency and Openness

The design and analysis plan for this study were not pre-registered. The materials and data for the Singapore sample are publicly accessible on Research box #3705 (https://researchbox.org/3705), while those for the US sample can be found on ICPSR (https://www.icpsr.umich.edu/), accessed on 7 November 2024. Additional resources such as analytic code, zero-order correlation matrices, a list of variables used for the study, and full results will be made available.

Analyses were conducted using R version 4.4.1 [51]. Data were handled using *dplyr* version 1.1.4 [52]. Descriptives were calculated using *psych* version 2.4.3 [53], while multilevel reliabilities were calculated using *lavaan* version 0.6–18 [54] with *semTools* version 0.5–6 [55]. Multilevel modelling was conducted using *lme4* version 1.1–35.5 [56] with *bobyqa* optimisation, with significance testing conducted through *lmerTest* version 3.1–3 [57]. Effect sizes in the form of standardised coefficients and 95% CIs were calculated using *effectsize* version 0.6.0.1 [58] with the method set to *pseudo*, and with datawizard version 0.12.2 [59]. Visualisations for results were produced using *ggplot2* version 3.5.1 [60] and *ggpubr* version 0.6.0 [61].

### 2.2. Design and Sample

The data for this study were drawn from two independent projects. One project examined young adults in Singapore [17,32,62,63,64] while the other project examined adults across the lifespan in the US [65,66,67,68]. By selecting these distinct age groups from diverse cultural contexts, this study aimed to explore potential confounding variables associated with age- or culture-specific differences in emotional experiences and cognitive functioning, ultimately contributing to a more robust and generalisable understanding of the observed relationships.

Both studies employed similar data collection methods. Initially, baseline data were gathered through self-administered surveys followed by data collection using a daily diary method. The Singapore sample completed their daily diary process over seven consecutive days through online self-administered surveys, while the US sample completed their daily diary process over eight consecutive days via telephone interviews. Informed consent was obtained from all participants prior to the start of data collection. The data collection procedures received approval from the Institutional Review Boards at Singapore Management University (for the Singapore sample) and the University of Wisconsin–Madison (for the US sample).

For the Singapore sample, data collection occurred between June and August 2021, with 253 participants providing 1721 daily observations (97% response rate). Based on previous research suggesting a minimum sample size of 250 for stable estimates [69], we aimed to enlist at least 250 participants. Demographic data and characteristics for the Singapore sample are summarised in Table 1.

For the US sample, baseline data were collected between May 2013 and November 2014, and daily diary data were collected between January 2017 and December 2019 for MIDUS 3. Baseline data were collected between November 2011 and September 2014, and daily diary data were collected between October 2012 and November 2014 for MIDUS Refresher 1. A total of 1726 participants contributed 12,722 daily observations across both datasets (92% response rate). The sampling process incorporated four subsamples to ensure a diverse and representative dataset, capturing a wide range of geographic groups within the US. Demographic data and characteristics for the US samples are summarised in Table 2.

### 2.3. Measures

#### 2.3.1. Baseline

**Demographic Variables.** In each sample, the demographic variables recorded included participant age (in years), sex (0 = *Female,* 1 = *Male*), main racial identity (Singapore sample: recoded into 0 = *Chinese* or 1 = *Non-Chinese* for participant anonymity; US sample: recoded into 0 = *White* or 1 = *Non-White* for participant anonymity), household income (Singapore sample, monthly: 1 = *less than 2000 SGD*, 2 = *2000 to 5999 SGD*, *3 = 6000 to 9999 SGD*, 4 = *10,000 to 14,999 SGD*, 5 = *15,000 to 19,999 SGD*, 6 = *more than 20,000 SGD*; US sample, annual: free response, top-coded at *300,000 USD*), and subjective social status within the community rated on a 10-point scale (where 1 is the lowest status and 10 is the highest status; [70]). For the US sample, marital status (recoded into 0 = *Married* or 1 = *Non-Married* for anonymity) and education level (1 = *No School/Grade School*, 2 = *Eighth Grade/Junior High School*, 3 = *Some High School*, 4 = *GED*, 5 = *Graduated from High School*, 6 = *1 to 2 years of College, no degree yet*, 7 = *3 or more years of College, no degree yet*, 8 = *Graduated from 2 year College, Vocational School, or Assoc. Deg.*, 9 = *Graduated from a 4 or 5 year College, or Bachelor’s Deg., 10 = Some Graduate School*, 11 = *Master’s Degree, 12 = PHD., ED.D., MD, DDS, LLB, LLD, JD, or other Professional Degree*) were also included.

#### 2.3.2. Day-Level Variables

**Daily Positive and Negative Affect.** In both samples, the Daily Distress Scale developed by Mroczek and Kolarz [71] was used to assess daily positive and negative affect. Participants rated the frequency of experiencing 13 positive emotions (e.g., satisfied, enthusiastic, etc.) and 14 negative emotions (e.g., hopeless, jittery, etc.) throughout a particular day on a five-point scale (0 = *None of the time* to 4 = *All of the time*). Daily positive affect scores were obtained by averaging the 13 items (Singapore sample: α_within_ = 0.94, α_between_ = 0.98; US sample: α_within_ = 0.86, α_between_ = 0.97). Daily negative affect scores were obtained by averaging the 14 items (Singapore sample: α_within_ = 0.89, α_between_ = 0.96; US sample: α_within_ = 0.78, α_between_ = 0.93). Scores were computed such that higher scores reflected higher daily positive or negative affect levels.

**Daily Cognitive Failures.** Daily cognitive failures was operationalised differently in the Singapore sample and in the US sample. Specifically, it was operationalised as a severity score for the Singapore sample but as a count of occurrences in the US sample.

In the Singapore sample, the Questionnaire for Cognitive Failures in Everyday Life [72] was used to assess the daily cognitive failures of each participant. This questionnaire included 13 items that described cognitive failures individuals might have experienced in the past 24 h (e.g., “Did you leave a task unfinished due to distraction(s) at any point today?”). Participants reported the frequency of each cognitive failure using a four-point scale (0 = *Never*, 1 = *Once*, 2 = *Twice*, or 3 = *Several times*), and the daily average score was calculated for each participant (tau-equivalent reliability, i.e., Cronbach’s α_within_ = 0.75, α_between_ = 0.95).

In the US sample, a checklist that evaluated everyday memory lapses [73] was used to assess each participant’s daily cognitive failures. The checklist included nine items related to cognitive failures that may have occurred for participants in the past 24 h (e.g., “forgetting to do an errand or chore” or “forgetting why you entered a room”). Participants indicated whether each cognitive failure occurred that day by selecting “Yes” (scored as 1) or “No” (scored as 0) for each item. Scores for daily cognitive failures were then calculated by summing the responses for each participant each day.

### 2.4. Analytical Plan

#### 2.4.1. General Overview

Given the nested structure of the data, where participants (Level 2) provided repeated observations across multiple days (Level 1), we employed multilevel modelling to address the dependency of daily data for each participant. Across both samples, we had the same overarching research question: What is the influence of positive affect and negative affect on cognitive failures, both at the between-person and within-person levels? To answer this question, we ran two-level models, with daily cognitive failures modelled as a function of daily positive and negative affect, adding demographic variables in subsequent models to test the robustness of this relationship. Based on the results presented in the next section (see “Inclusion of Day as a Predictor”), we also included or excluded day as a fixed and/or random predictor in all the models in order to de-bias the estimates of interest where necessary. Analyses were run to first obtain unstandardised coefficients, and corresponding standardised coefficients were then obtained through re-estimation of the same models.

For the Singapore sample, linear regressions were conducted as the severity of daily cognitive failures was measured as a continuous variable. The severity score was also standardised when obtaining standardised coefficients. That is, obtained standardised regression coefficients would correspond to standard deviation units (e.g., a coefficient of 0.5 for positive affect at the within-person level would imply that for every 1 *SD* increase in positive affect, the severity of cognitive failures would increase by 0.5 *SD* at the within-person level).

For the US sample, Poisson loglinear regressions were conducted as daily cognitive failures was measured as a count variable. The count was not standardised when obtaining standardised coefficients due to its inherent meaning as a count (i.e., how many cognitive failures occurred). That is, obtained standardised regression coefficients would correspond to standard deviation units in the predictor but not in the outcome (e.g., a coefficient of 0.5 for positive affect at the within-person level would imply that for every 1 *SD* increase in positive affect, the number of cognitive failures would increase by 0.5 at the within-person level).

#### 2.4.2. Inclusion of Day as a Predictor

Prior to the main analyses, we assessed the fit of three alternative multilevel regression models that differed in their modelling of day (i.e., time in the study) so as to identify the best-fitting model for the current data. We conducted this examination separately for the Singapore sample (using a linear model) and for the US sample (using a Poisson loglinear model). For brevity, we present only the linear models in this section.

Initially, we estimated a null model (Model A) by including only a fixed intercept γ_00_ and a random intercept μ_0*i*_, without the day term, as follows:

Level 1:

(Daily cognitive failures)*_di_* = B_0*i*_ + ε*_di_*

Level 2:

B_0*i*_ = γ_00_ + μ_0*i*_

Subsequently, we incorporated day as a fixed predictor with coefficient γ_30_ to examine its relation to cognitive failures, resulting in the following second model (Model B):

Level 1:

(Daily cognitive failures)*_di_* = B_0*i*_ + B_3*i*_(day)*_di_* + ε*_di_*

Level 2:

B_0*i*_ = γ_00_ + μ_0*i*_

B_3*i*_ = γ_30_

Lastly, we specified a third model (Model C), which included day as both a fixed predictor (with coefficient γ_30_) and a random predictor (with coefficient μ_3*i*_), allowing for individual variation in the effect of day across each participant *i*, as follows:

Level 1:

(Daily cognitive failures)*_di_* = B_0*i*_ + B_3*i*_(day)*_di_* + ε*_di_*

Level 2:

B_0*i*_ = γ_00_ + μ_0*i*_

B_3*i*_ = γ_30_ + μ_3*i*_

Consistent with recommended practices of model comparison and selection outlined in the literature [74,75,76,77,78], the model fit was evaluated using two widely used information criteria—the Akaike Information Criterion (AIC) and the Bayesian Information Criterion (BIC). The model with the lowest AIC and BIC values was selected as the best-fitting model, offering the most parsimonious and accurate representation of the data while accounting for potential true variability across participants.

#### 2.4.3. Unadjusted Model

To separate the within-person and between-person relationships in our multilevel models, person-mean centring was carried out for daily positive affect and daily negative affect [79]. Specifically, each person’s score for each day was decomposed into a person-mean component (Level 2) and a deviation component (Level 1). Both daily positive and negative affect deviations were added as simultaneous predictors at Level 1, while each participant’s average positive and negative affect were reintroduced at Level 2.

The linear model is specified as follows, with γ_01_ and γ_02_ representing the between-person parameter of interest, and γ_10_ and γ_20_ representing the within-person parameter of interest, respectively:

Level 1:

(Daily cognitive failures)*_di_* = B_0*i*_ + B_1*i*_(deviation of positive affect)*_di_* + B_2*i*_(deviation of negative affect)*_di_* + B_3*i*_(day)*_di_* + ε*_di_*

Level 2:

B_0*i*_ = γ_00_ + γ_01_(average of positive affect)*_i_* + γ_02_(average of negative affect)*_i_* + μ_0*i*_
B_1*i*_ = γ_10_ + μ_1*i*_
B_2*i*_ = γ_20_ + μ_2*i*_
B_3*i*_ = γ_30_ + μ_3*i*_

The Poisson loglinear model is specified similarly at Level 2, with the Level 1 equation modified to the following:

Level 1:

*log*(Daily cognitive failures)*_di_* = B_0*i*_ + B_1*i*_(deviation of positive affect)*_di_* + B_2*i*_(deviation of negative affect)*_di_* + B_3*i*_(day)*_di_* + ε*_di_*

In the case where day did not have to be included as a random effect, μ_3*i*_ was not estimated and fixed at 0. If day did not have to be included at all (i.e., not even as a fixed effect), the γ_30_ term was also not estimated and fixed at 0.

#### 2.4.4. Adjusted Model

To de-bias the between-person estimates (i.e., γ_01_ and γ_02_) and the ensure robustness of the previous findings, an adjusted model for each sample was run, incorporating demographic variables that previous studies identified as affecting cognitive health [80,81,82,83]. Continuous variables (Singapore and US sample: age, household income, and subjective social status; US sample only: education level) were grand-mean centred while binary variables (Singapore and US samples: race, where 0 = *Majority race* i.e., *Chinese* or *White*, and 1 = *Minority race*, and sex, where 0 = *Female* and 1 = *Male*; US sample only: marital status, where 0 = *Married* and 1 = *Non-Married*) were dummy coded. For the US sample, the model included additional demographic variables (i.e., marital status and education level) as the US sample included adults from a broader age range and more diverse demographic backgrounds.

## 3. Results

### 3.1. Inclusion of Day as a Predictor

For both the Singapore and US sample, a comparison of the models’ AIC and BIC values indicated that Model C, which included day as both a fixed and random predictor, provided the best fit overall (Table 3). This supported the inclusion of day as both a fixed and random predictor in subsequent analyses.

### 3.2. Singapore Sample

**Figure 1:**
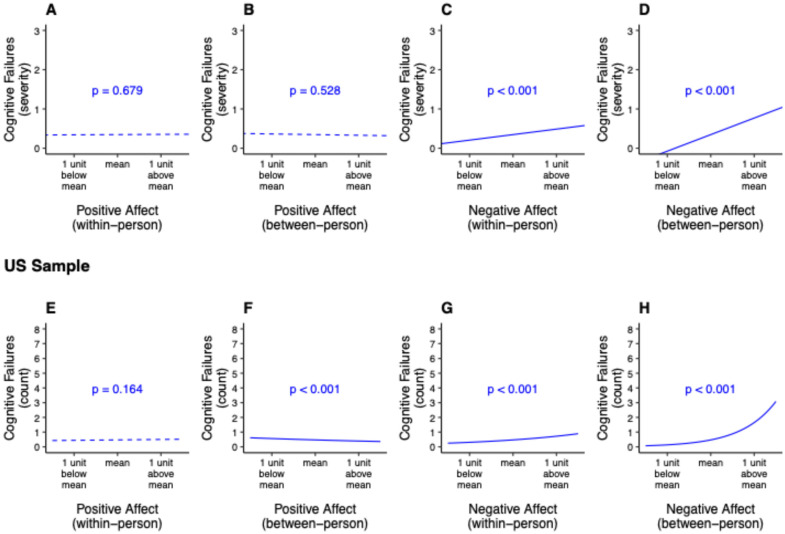
(**A**–**H**) Summary of adjusted results for between- and within-person associations of positive and negative affect with cognitive failures for the Singapore and US samples. In the Singapore sample, *N*_participants_ = 253 and *N*_observations_ = 1721. In the US sample, *N*_participants_ = 1726 and *N*_observations_ = 12,722. Slopes and *p*-values depicted are drawn from fully adjusted models. A solid line refers to a significant association, while a dashed line refers to a non-significant association.

Positive affect was not significantly associated with cognitive failures at the within-person level (unadjusted: β = 0.01, 95% CI = [−0.04, 0.07], γ_10_ = 0.006, *SE* = 0.01, *p* = 0.683; adjusted: β = 0.01, 95% CI = [−0.04, 0.07], γ_10_ = 0.006, *SE* = 0.01, *p* = 0.679; Figure 1A) nor at the between-person level (unadjusted: β = −0.04, 95% CI = [−0.15, 0.07], γ_01_ = −0.02, *SE* = 0.03, *p* = 0.484; adjusted: β = −0.04, 95% CI = [−0.15, 0.08], γ_01_ = −0.02, *SE* = 0.03, *p* = 0.528; Figure 1B).

In comparison, negative affect was significantly associated with cognitive failures to a medium extent at the within-person level (unadjusted: β = 0.21, 95% CI = [0.14, 0.27], γ_20_ = 0.14, *SE* = 0.02, *p* < 0.001; adjusted: β = 0.21, 95% CI = [0.14, 0.27], γ_20_ = 0.14, *SE* = 0.02, *p* < 0.001: Figure 1C) and to a very large extent at the between-person level (unadjusted: β = 0.58, 95% CI = [0.47, 0.69], γ_02_ = 0.43, *SE* = 0.04, *p* < 0.001; adjusted: β = 0.57, 95% CI = [0.46, 0.69], γ_02_ = 0.42, *SE* = 0.04, *p* < 0.001; Figure 1D). At both the within- and between-person levels, the pattern was such that as negative affect increased, the severity of cognitive failures also increased. That is, comparing each person to themselves, on days with higher levels of negative affect, the severity of cognitive failures was also higher. In addition, people with higher levels of negative affect in general (compared to people with lower levels of negative affect in general) faced a higher severity of cognitive failures in general.

### 3.3. US Sample

Consistent with the findings in the Singapore sample, positive affect was not significantly associated with daily cognitive failures at the within-person level (unadjusted: β = 0.02, 95% CI = [−0.01, 0.05], γ_10_ = 0.06, *SE* = 0.04, *p* = 0.157; adjusted: β = 0.02, 95% CI = [−0.01, 0.05], γ_10_ = 0.06, *SE* = 0.05, *p* = 0.164; Figure 1E). However, in contrast to the findings for the Singapore sample, positive affect showed a significant association with cognitive failures to a small extent at the between-person level (unadjusted: β = −0.11, 95% CI = [−0.17, −0.05], γ_01_ = −0.15, *SE* = 0.04, *p* < 0.001; adjusted: β = −0.13, 95% CI = [−0.18, −0.07], γ_01_ = −0.18, *SE* = 0.04, *p* < 0.001; Figure 1F). The pattern was such that people with higher levels of positive affect in general (compared to people with lower levels of positive affect in general) faced a lower occurrence of cognitive failures on average.

In line with the findings from the Singapore sample, negative affect was significantly associated with cognitive failures to a very small extent at the within-person level (unadjusted: β = 0.08, 95% CI = [0.06, 0.11], γ_20_ = 0.43, *SE* = 0.07, *p* < 0.001; adjusted: β = 0.08, 95% CI = [0.06, 0.11], γ_20_ = 0.43, *SE* = 0.07, *p* < 0.001; Figure 1G) and to a medium extent at the between-person level (unadjusted: β = 0.28, 95% CI = [0.23, 0.34], γ_02_ = 1.17, *SE* = 0.11, *p* < 0.001; adjusted: β = 0.30, 95% CI = [0.25, 0.35], γ_02_ = 1.25, *SE* = 0.11, *p* < 0.001; Figure 1H). At both the within- and between-person levels, the pattern was such that as negative affect increased, the occurrence of cognitive failures also increased. That is, comparing each person to themselves, on days with higher levels of negative affect, the occurrence of cognitive failures was also higher. In addition, people with higher levels of negative affect in general (compared to people with lower levels of negative affect in general) faced a higher occurrence of cognitive failures in general.

## 4. Discussion

This study aimed to investigate the relationship between affect and cognitive failures through two daily diary studies; Study 1 included a sample of 253 young adults in Singapore over seven days, while Study 2 consisted of 1726 older adults in the United States over eight days. The findings revealed discrepancies in the relationship between daily positive affect and daily cognitive failures, whereas a consistent relationship was observed between daily negative affect and cognitive failures. These relationships remained significant even after controlling for demographic covariates known to influence cognitive functioning [80,81,82,83]. This study extends our understanding of the affective mechanisms underlying daily cognitive failures and highlights the complexity of affective influences on cognitive processes.

Our initial hypothesis posited that daily and average positive affect would be positively correlated with cognitive failures, with the expectation that positive affect would broaden attention and thus increase cognitive lapses. However, the results did not fully support this hypothesis. Instead, the findings suggest that positive affect may narrow attentional focus, thereby reducing cognitive failures. For the Singapore sample, no significant association was found between daily positive affect and daily cognitive failures. In contrast, for the US sample, although no significant within-person relationship was observed, a higher general level of positive affect was significantly associated with fewer occurrences of cognitive failures at the between-person level. We speculate that the null findings for within-person relationships for both samples may be attributed to a potential “push and pull” effect; while positive affect can broaden attention and potentially impair cognitive performance, positive affect has also been linked to enhanced executive functioning [84,85,86], which could mitigate cognitive lapses. The interplay between these effects might help explain the observed null relationship between positive affect and cognitive failures at the within-person level.

Meanwhile, in the US sample, the significant between-person relationship suggests that individuals with generally higher levels of positive affect tend to experience fewer cognitive lapses, while the between-person relationship in the Singapore sample was not significant. The lack of significant findings in the Singapore sample may be influenced by various factors including cultural differences in emotional expression and emotion regulation [87,88,89]. For instance, research indicates that individuals from Asian cultures often experience more suppression of emotional expression compared to their Western counterparts [90], which may reduce the positive effect of positive emotions on cognitive failures [91]. This difference in emotional regulation strategies suggests that cultural context may play a key role in shaping the relationship between affect and cognitive performance. In many Western cultures, positive emotional expression is encouraged and viewed as beneficial, whereas Asian cultures, such as Singapore, tend to emphasise emotional restraint to maintain social harmony and self-control [92,93]. Future research should explore the cultural dimensions of emotion regulation and their moderating influence on the relationship between affect and cognitive failures through collecting cultural data. Additionally, as emotional regulation can also differ across the lifespan, we acknowledge that the impact of emotional affect on cognitive failures may vary between younger and older adults. Specifically, age-related changes in emotional processing may affect how individuals respond to positive emotions. For instance, older adults may demonstrate more developed emotion regulation capacity compared to younger adults [94,95], which might allow positive emotions to have a more beneficial effect on cognitive performance [96]. This may also contribute to the significant negative association between positive affect and cognitive failures observed in the US sample, which was not significant in the Singapore sample. Taken together, these findings suggest a more complex relationship between positive affect and cognitive failures. While our results indicate that positive affect may enhance cognitive function, the existing literature also demonstrates that positive affect can broaden attentional focus in certain contexts, which may not always align with improvements in cognitive performance [85,97]. Moreover, not all forms of positive affect, such as gratitude, are consistently linked to enhanced cognitive function [98]. Future research should investigate the specific types of positive emotions and their distinct impacts on daily cognitive failures.

Our initial hypothesis posited that daily and average negative affect would be negatively correlated with cognitive failures, based on the expectation that negative affect would narrow attention and thereby decrease cognitive lapses. However, the results did not fully support this hypothesis. Instead, negative affect consistently demonstrated a positive association with cognitive failures at both the within-person and between-person levels across both samples, suggesting that negative affect may broaden attention, potentially leading to increased cognitive lapses. While research indicates that negative affect can narrow attentional focus and reduce distractions [25,28], this effect may not be sufficient to counterbalance the detrimental impact of heightened emotional distress associated with negative affectivity. The current findings align with existing literature that links negative emotions, such as stress, anxiety, and depression, with increased cognitive lapses, particularly in domains such as attention, working memory, and verbal fluency [99,100,101]. Specifically, in the Singapore sample, individuals reporting higher daily levels of negative affect experienced more severe cognitive failures. Similarly, in the US sample, higher levels of negative affect were associated with a greater frequency of cognitive lapses. This is consistent with previous research demonstrating that negative affective experiences demand greater cognitive resources, leading to attentional deficits that contribute to cognitive lapses [21,102]. This effect could be attributed to the tendency of negative emotions to promote mind-wandering [103,104,105]. Negative affect, often associated with stress [106,107], can cause cognitive lapses by unintentionally shifting attention towards unrelated thoughts about daily stressors (i.e., stress-induced mind-wandering), thereby impairing task focus [37,108]. Moreover, negative affect is frequently associated with conditions such as depression and anxiety [109,110,111], which have been shown to impair cognitive functions by heightening sensitivity to threat-related stimuli, as outlined in Attentional Control Theory [112,113,114], thus removing attentional focus from daily tasks and increasing the potential for cognitive failures [115].

Furthermore, the effect of negative affect appeared to be stronger between individuals than within individuals for both samples. This suggests that individual differences in chronic negative affective states are more strongly linked to cognitive lapses than fluctuations in negative emotions within the same person over time. This pattern aligns with prior studies demonstrating the adverse effects of chronic negative emotions on cognitive performance [32,43]. For example, research has shown that individuals with consistently higher levels of negative affect are more prone to cognitive failures, such as memory lapses and attentional errors, compared to those with lower average negative affect [99,103]. These findings underscore the critical importance of addressing chronic negative affective states to mitigate their harmful impact on cognitive functioning.

The current study sets itself apart from prior research on the affective aspect of cognitive failures by employing a daily diary methodology, which allows for a real-time assessment of affective fluctuations and daily cognitive failures [47,48]. Thus, this method provides a more ecologically valid understanding of how affective states influence cognitive lapses in naturalistic settings by reducing the time lag for recall [116,117,118,119]. Moreover, by examining both within-person and between-person effects, this study provides a more comprehensive understanding of how affective experiences influence cognitive functioning. While previous research has predominantly examined between-person relationships, this work extends these findings by exploring the importance of considering both stable individual differences and temporal fluctuations in the influence of affective experiences on cognitive lapses.

Several limitations of this study must be acknowledged. First, the affective measures employed primarily assess general affective states, potentially overlooking the full spectrum of affective experiences. Given that emotions encompass both valence and arousal [120,121], it would be beneficial for future research to incorporate more specific affective scales, such as the PANAS-X scale [122], which includes dimensions like fatigue and surprise, or scales based on the circumplex model of affect [123], which captures additional emotions like tension and unhappiness. Such measures would provide a more comprehensive understanding of how various affective states contribute to cognitive failures. Secondly, the discrepancy in the operationalisation of daily cognitive failures between the two samples (i.e., severity vs. count) may have complicated direct comparisons. Future research would benefit from standardizing the measures of cognitive failures across samples to ensure consistency, thereby facilitating more meaningful comparisons and strengthening the robustness of research in this area. Next, while the daily diary method helps mitigate recall bias, the reliance on self-reported data remains an inherent limitation due to its subjective nature. Future studies should consider integrating objective measures of cognitive performance [124] to complement self-reported data and provide more reliable insights into the cognitive processes underlying the observed relationships. Additionally, while within-person associations help control for individual differences, potential confounding factors, such as time-varying confounds, remain unaddressed. Therefore, the findings should not be interpreted as establishing causality. There is also the possibility of reverse causation, whereby cognitive failures may influence daily affective states [125,126,127]. Finally, while the study sampled participants from Singapore and the US, the samples were predominantly from higher socioeconomic backgrounds with similar educational levels. This could limit the generalisability of the findings to more diverse or representative populations. Meanwhile, building on a growing body of research, interventions that utilise music could be valuable tools for influencing emotional states and enhancing cognitive performance [128,129,130,131]. Future studies may investigate how music-based interventions can specifically address the impact of affective experiences on cognitive failures, exploring both immediate and long-term effects on cognitive functioning.

## 5. Conclusions

This study revealed distinct patterns in the relationships between affect and daily cognitive failures across the Singapore and US samples. Overall, positive affect showed no significant associations with daily cognitive failures in the Singapore sample and only a between-person negative association in the US sample, while negative affect consistently exhibited significant positive relationships with daily cognitive failures across both within- and between-person levels in both samples. These findings provide valuable insights into the affective mechanisms influencing cognitive failures, highlighting the predictive potential of negative affect for the occurrence of daily cognitive lapses based on affective data. This relationship is particularly important for older adults, given the heightened impact of cognitive failures on their daily functioning, health, and well-being [132]. Negative affect has been linked to worsened cognitive performance and instrumental activities of daily living (IADLs) in older adults, while positive affect has been shown to potentially buffer these effects and improve cognitive function [133,134], impacting their daily functioning. These findings highlight the need to consider emotional affect when examining cognitive failures, particularly in older populations. Furthermore, future research should aim to address the study’s limitations by incorporating more comprehensive measures of affect and integrating objective assessments of cognitive failures to better elucidate the mechanisms linking affective states to cognitive functioning.

## Figures and Tables

**Table 1:** Descriptive Statistics of the Singapore Sample.

Variable	*N*	*M* (*SD*) or %	Observed Range	Theoretical Range
**Participant Level** ^a^				
*Demographics*				
Age	253	22.11 (1.63)	19–29	
Sex (% Female)	253	76.68%		
Race (%)	253			
Chinese	190	75.10%		
Malay	12	4.74%		
Indian	26	10.28%		
Others	25	9.88%		
Monthly household income	253	3.00 (1.43)	1–6	1–6
Subjective social status	253	6.11 (1.25)	2–10	1–10
*Average of day-level variables*				
Average positive affect	253	1.89 (0.72)	0.00–3.97	0.00–4.00
Average negative affect	253	0.57 (0.45)	0.00–2.29	0.00–4.00
Average cognitive failures	253	0.34 (0.35)	0.00–2.87	0.00–4.00
**Day Level** ^b^				
Daily positive affect	1721	1.90 (0.91)	0.00–4.00	0.00–4.00
Daily negative affect	1721	0.57 (0.61)	0.00–3.93	0.00–4.00
Daily cognitive failures (severity)	1721	0.33 (0.44)	0.00–3.00	0.00–4.00

Note. ^a^ *N*s refer to number of participants. ^b^ *N*s refer to number of observations.

**Table 2:** Descriptive Statistics of the US Sample.

Variable	*N*	*M* (*SD*) or %	Observed Range	Theoretical Range
**Participant Level** ^a^				
*Demographics*				
Age	1726	56.22 (13.51)	25–90	
Sex (% female)	1726	55.04%		
Race (%)	1726			
White	1520	88.06%		
Black	74	4.29%		
Other	132	7.65%		
Marital status (% Married)	1726	66.98%		
Education level	1726	7.93 (2.41)	1–12	1–12
Annual household income (in units of 10,000)	1726	8.98 (6.87)	0–30	0–30
Subjective social status	1726	6.43 (1.83)	1–10	1–10
*Average of day-level variables*				
Average positive affect	1726	2.61 (0.71)	0.21–4.00	0.00–4.00
Average negative affect	1726	0.19 (0.24)	0.00–2.86	0.00–4.00
Average cognitive failures	1726	0.71 (0.82)	0.00–7.00	0.00–9.00
**Day Level** ^b^				
Daily positive affect	12,722	2.61 (0.79)	0.00–4.00	0.00–4.00
Daily negative affect	12,722	0.19 (0.30)	0.00–3.43	0.00–4.00
Daily cognitive failures (count)	12,722	0.69 (1.07)	0.00–8.00	0.00–9.00

Note. ^a^ *N*s refer to number of participants. ^b^ *N*s refer to number of observations.

**Table 3:** Model Fit Indices of the Various Models.

	Singapore (*N*_participants_ = 253, *N*_observations_ = 1721)		US (*N*_participants_ = 1726, *N*_observations_ = 12,722)	
Model	AIC	BIC	AIC	BIC
Model A (null model)	1165	1182	26,092	26,107
Model B (day as fixed)	1140	1162	25,856	25,878
Model C (day as fixed and random)	1107	1140	25,757	25,794

## Data Availability

Publicly available datasets were analysed in this study. The materials and data for the Singapore sample are publicly accessible on Research box #3705 (https://researchbox.org/3705), while those for the US sample can be found on ICPSR (https://www.icpsr.umich.edu/), accessed on 7 November 2024.
